# DNA methylation as a predictor of fetal alcohol spectrum disorder

**DOI:** 10.1186/s13148-018-0439-6

**Published:** 2018-01-12

**Authors:** Alexandre A. Lussier, Alexander M. Morin, Julia L. MacIsaac, Jenny Salmon, Joanne Weinberg, James N. Reynolds, Paul Pavlidis, Albert E. Chudley, Michael S. Kobor

**Affiliations:** 1 Department of Medical Genetics, Centre for Molecular Medicine and Therapeutics, British Columbia Children’s Hospital Research Institute, University of British Columbia, Vancouver, British Columbia Canada; 2 Department of Cellular and Physiological Sciences, Life Sciences Institute, University of British Columbia, Vancouver, British Columbia Canada; 3 Department of Pediatrics and Child Health, Faculty of Medicine, University of Manitoba, Winnipeg, Manitoba Canada; 4 Department of Biochemistry and Medical Genetics, Faculty of Medicine, University of Manitoba, Winnipeg, Manitoba Canada; 5 Department of Biomedical and Molecular Sciences, Centre for Neuroscience Studies, Queen’s University, Kingston, Ontario Canada; 6 Michael Smith Laboratories, University of British Columbia, Vancouver, British Columnbia Canada; 7 Human Early Learning Partnership, University of British Columbia, Vancouver, British Columbia Canada; 8 Department of Psychiatry, University of British Columbia, Vancouver, British Columbia Canada

**Keywords:** Fetal alcohol spectrum disorder, Epigenetics, DNA methylation, Biomarkers, Neurodevelopmental disorders

## Abstract

**Background:**

Fetal alcohol spectrum disorder (FASD) is a developmental disorder that manifests through a range of cognitive, adaptive, physiological, and neurobiological deficits resulting from prenatal alcohol exposure. Although the North American prevalence is currently estimated at 2–5%, FASD has proven difficult to identify in the absence of the overt physical features characteristic of fetal alcohol syndrome. As interventions may have the greatest impact at an early age, accurate biomarkers are needed to identify children at risk for FASD. Building on our previous work identifying distinct DNA methylation patterns in children and adolescents with FASD, we have attempted to validate these associations in a different clinical cohort and to use our DNA methylation signature to develop a possible epigenetic predictor of FASD.

**Methods:**

Genome-wide DNA methylation patterns were analyzed using the Illumina HumanMethylation450 array in the buccal epithelial cells of a cohort of 48 individuals aged 3.5–18 (24 FASD cases, 24 controls). The DNA methylation predictor of FASD was built using a stochastic gradient boosting model on our previously published dataset FASD cases and controls (GSE80261). The predictor was tested on the current dataset and an independent dataset of 48 autism spectrum disorder cases and 48 controls (GSE50759).

**Results:**

We validated findings from our previous study that identified a DNA methylation signature of FASD, replicating the altered DNA methylation levels of 161/648 CpGs in this independent cohort, which may represent a robust signature of FASD in the epigenome. We also generated a predictive model of FASD using machine learning in a subset of our previously published cohort of 179 samples (83 FASD cases, 96 controls), which was tested in this novel cohort of 48 samples and resulted in a moderately accurate predictor of FASD status. Upon testing the algorithm in an independent cohort of individuals with autism spectrum disorder, we did not detect any bias towards autism, sex, age, or ethnicity.

**Conclusion:**

These findings further support the association of FASD with distinct DNA methylation patterns, while providing a possible entry point towards the development of epigenetic biomarkers of FASD.

**Electronic supplementary material:**

The online version of this article (10.1186/s13148-018-0439-6) contains supplementary material, which is available to authorized users.

## Background

Fetal alcohol spectrum disorder (FASD) is a leading preventable cause of developmental disability, with a North American prevalence currently estimated at 2–5% [1–3]. FASD presents through a wide spectrum of phenotypes, ranging from growth deficits and physical abnormalities to cognitive and behavioral deficits, as well as motor and sensory impairments, immune dysfunction, and increased vulnerability to mental health problems in adulthood [4–6]. On the most severe end of the spectrum lies fetal alcohol syndrome (FAS), which is characterized by growth retardation, a distinct set of facial dysmorphisms, and central nervous system abnormalities [7, 8]. By contrast, Alcohol-Related Neurodevelopmental Disorder (ARND) describes the less visible and largest group within the spectrum, where individuals with confirmed alcohol exposure during pregnancy show primarily behavioral, adaptive, and/or cognitive abnormalities without obvious facial dysmorphisms [9]. Of note, individuals across the spectrum show cognitive and behavioral deficits, which can be as serious in those without any physical features as in those with full FAS [10].

Although children with FAS are often diagnosed in infancy or in early life, FASD in general has proven difficult to identify, particularly in the absence of the overt facial features characteristic of FAS. As such, many individuals with FASD are not identified until they reach school age, where they begin to struggle with increased social pressure and cognitive challenges [11]. However, early cognitive and behavioral interventions may potentially attenuate some of the deficits associated with FASD and improve the long-term outcomes of these individuals [12]. As early diagnosis is a strong predictor of positive outcome, early screening tools are necessary to help identify at-risk children at a young age and potentially buffer some of the deficits associated with prenatal alcohol exposure (PAE) [13, 14].

While self-report methods are most commonly used for assessing PAE and a child’s risk for FASD, these are not always accurate and can underestimate alcohol consumption during pregnancy [15–17]. Over the past decades, various biomarkers of alcohol exposure have been developed to complement self-report measures, focusing primarily on the direct or indirect products of ethanol metabolism, which can be measured in biological specimens from both the mother and infant [18]. Although these biomarkers are very sensitive to alcohol exposure, they present a number of limitations when attempting to determine whether prenatal alcohol exposure has occurred or to gain insight into the biological underpinnings of alcohol-induced deficits and the developmental profiles associated with FASD. For example, many of these biomarkers have short windows of detection (e.g., urine, blood, plasma) or are limited by specimen availability (e.g., placenta, meconium), making them useful for identification of alcohol exposure around the time of parturition, but not in infants and children over the course of development [19]. As such, objective and persistent measures are needed to aid in the screening and diagnosis of children at risk for FASD.

Epigenetic marks are now emerging as potential biomarkers or signatures of early-life exposures. Broadly defined, epigenetics refers to modifications of DNA and its regulatory components, including chromatin and non-coding RNA, that potentially modulate gene transcription without changing underlying DNA sequences [20–22]. In addition to their role in the regulation of cellular processes, these may also bridge environmental factors and genetic regulation to capture a lasting signature of early life exposures. In particular, DNA methylation is emerging as a candidate biomarker for environmental exposures and disease. Typically found on the cytosine residues of cytosine-guanine dinucleotides (CpG), this epigenetic mark is both stable over time and dynamic in response to environmental factors [23]. Several pre- and postnatal environmental influences have been associated with altered DNA methylation patterns, hinting at possible malleability by early-life environments and suggesting a potential utility as biomarkers [24, 25]. For example, prenatal exposure to cigarette smoke is associated with lasting alterations to DNA methylation patterns, which are now being used as biomarkers of cigarette smoke exposure in infants [26].

While in its infancy in relation to FASD, epigenetic biomarkers show promise for early screening of at-risk individuals, as the DNA methylome retains a lasting signature of prenatal alcohol exposure in both the central nervous system and peripheral tissues (reviewed in [27]). Numerous studies performed in animal and cell culture models have identified both short-term and persistent alterations to DNA methylation patterns following PAE. Although some of these models reflect supra-physiological levels of alcohol exposure or display modest effect sizes in response to PAE, the findings from these pre-clinical models suggest the possibility that PAE may directly influence epigenetic patterns and that these may play a role in PAE-induced deficits [27–33]. By contrast, fewer studies have investigated DNA methylation patterns in individuals with FASD. More targeted methods identified differences in DNA methylation levels in the promoter region of *Drd4* in a large Australian cohort of children exposed to alcohol during breastfeeding [34]. Others have employed discovery-driven approaches, assessing genome-wide DNA methylation patterns in case-control studies of FASD. The first of these came from a small cohort of children, where slight differences in DNA methylation patterns within the protocadherin (PCDH) gene clusters reported with a rather modest significance threshold [35]. Recently, we analyzed DNA methylation profiles in a large cohort of children with FASD recruited by NeuroDevNet (NDN), a Canadian Networks of Centres of Excellence, where we identified a signature of 658 differentially methylated CpGs [36]. Although few results have been validated across different cohorts, these findings set the stage for broader applications of DNA methylation in the context of FASD, creating a framework upon which to build future epigenomic studies of FASD.

To validate the findings from our previous DNA methylation study, we assessed the genome-wide DNA methylation profiles of buccal epithelial cells (BEC) from an independent cohort of 24 individuals with FASD, aged 3.5–18, and 24 typically developing controls, aged 5–17. Given that our initial study provided a framework for genome-wide assessment of DNA methylation patterns in individuals with FASD, we used the findings from the NDN study as a foundation for the identification of replicable epigenetic differences associated with FASD. Notably, nearly 25% of statistically significant associations from the NDN cohort were validated in this new cohort at a false-discovery rate (FDR) < 0.05 [37]. In addition to the validation analyses, we also assessed whether DNA methylation profiles could be used to identify individuals with FASD, generating a classification algorithm that uses DNA methylation levels to accurately predict FASD status. Taken together, these results suggested that there were replicable differences in DNA methylation patterns between individuals with FASD and controls, which could potentially contribute to the development of a screening tool for at-risk children.

## Methods

### The Kids Brain Health Network cohort of children with FASD

The present cohort was collected as a replication study by Kids Brain Health Network (KBHN), formerly NeuroDevNet, and is hereby referred to as the KBHN cohort [38]. Ethics for this study were reviewed and approved by the “Children’s and Women’s Research Ethics Board – Clinical” (H10-01149). All experimental procedures were reviewed and approved by the University of Manitoba and the University of British Columbia. Written informed consent was obtained from a parent or legal guardian, and assent was obtained from each child before study participation. The clinics used previously described guidelines for the diagnosis of FASD [39]. Children with FASD and typically developing controls were recruited from the Manitoba FASD diagnostic clinic in Winnipeg, Manitoba, Canada. Briefly, buccal epithelial cell (BEC) samples were collected for DNA methylation analysis from 25 FASD and 26 age- and sex-matched control children aged between 3.5 and 18, prior to pre-processing (Table 1). BECs were collected using the Isohelix buccal swabs and Dri-Capsule (Cell Projects Ltd., Kent, UK). To collect buccal cells, the swab was inserted into the participants’ mouth and rubbed firmly against the inside of the left cheek for 1 min. The swab was then placed into a sterile tube with a Dri-Capsule and the tube sealed. An identical procedure was followed for the right cheek. Participants did not have any dental work performed 48 h prior to collection, and no food was consumed less than 60 min prior to collection to avoid contamination.

**Table 1:** Characteristics of the NeuroDevNet II FASD cohort

	FASD cases	Controls
*N*	24	24
ARND	18	
Partial FAS	6	
FAS	1	
FASD	1	
Age (years)		
Range	3.5–18	5–17
Mean	9.1	11.6
Sex		
Female	9	13
Male	15	11
Self-declared ethnicity		
Caucasian	4 (2)^a^	22
First Nations	17 (20)^a^	1
Asian	1 (0)^a^	1
Not reported	2	0
Caregiver status		
Biological parents	7	24
Biological grandparents	3	0
Adopted/legal guardian	8	0
Foster care	6	0

^a^Including mixed lineage First Nations

### DNA methylation 450K assay

DNA was extracted from BECs using the Isohelix DNA isolation kit (Cell Projects, Kent, UK). Seven hundred fifty nanograms of genomic DNA was subjected to bisulfite conversion using the Zymo EZ DNA Methylation Kit (Zymo Research, Irvine, California), which converts DNA methylation information into sequence base differences by deaminating unmethylated cytosines to uracil while leaving methylated cytosines unchanged. One hundred sixty nanograms of converted DNA was applied to the HumanMethylation450 BeadChip array from Illumina (450K array), which enables the simultaneous quantitative measurement of 485,512 CpG sites across the human genome, following the manufacturer’s instructions. Chips were scanned on an Illumina HiScan, with the 51 samples run in two batches and each containing a similar number of FASD and control samples, randomly distributed across the chips. Two pairs of technical replicates were also included and showed a Pearson correlation coefficient *r* > 0.994 in both cases, highlighting the technology’s reproducibility on our in-house platform. Inter-sample correlations ranged from 0.926–0.99.

### DNA methylation data quality control and normalization

The raw DNA methylation data were subjected to a rigorous set of quality controls, first of the samples, and then of the probes. Of the 51 initial samples, 3 were removed from the final dataset based on poor quality data, which was identified through skewed internal controls and/or > = 5% of probes with a detection *p* value > 0.05 (2 controls and 1 FASD). Next, probes were removed from the dataset according to the following criteria: (1) probes on X and Y chromosomes (*n* = 11,648), (2) SNP probes (*n* = 65), (3) probes with bead count < 3 in 10% of samples (*n* = 726), (4) probes with 10% of samples with a detection *p* value > 0.01 (*n* = 11,864), and (5) probes with a polymorphic CpG and non-specific probes (*N* = 19,337 SNP-CpG and 10,484 non-specific probes) [40]. A final filtering step was performed to set the methylation values to NA for any remaining probe-sample pair where bead count < 3 or detection *p* value > 0.01. Data normalization was performed using the SWAN method on the final dataset, composed of 48 samples (24 FASD and 24 controls) and 431,544 probes [41]. Finally, batch effects (chip number and chip position) were removed using the ComBat function from the *SVA* package in R [42]. Statistical analyses were performed using on ComBat-corrected *M* values, which represent the log2 ratio of methylated/unmethylated, where negative values indicate less than 50% methylation and positive values indicate more than 50% methylation [43]. Percent methylation differences (beta-values) were used in graphical representations of the data and indicate the percentage of methylation calculated by methylated/(methylated + unmethylated), ranging from 0 (fully unmethylated) to 1 (fully methylated).

### Differential methylation analysis and validation of NeuroDevNet (NDN) findings

Cell type deconvolution was performed to assess the proportions of CD14, CD34, and buccal epithelial cells in each sample using DNA methylation levels at CpGs highly correlated with these cell types [44]. Surrogate variable analysis (SVA) was also performed on ComBat-corrected, normalized data using the *SVA* package in R to identify surrogate variables (SVs) representative of unwanted heterogeneity [42]. Using DNA methylation data from all 48 samples, SVA identified 6 SVs not associated with clinical status (FASD vs control). As these were partially associated with known covariates, such as cell type proportions and age, the SVs were included in the linear regression analysis to account for their effects. More specifically, linear modeling was performed on the 648 differentially methylated probes identified in the initial NDN study and found in the present dataset using the *limma* package in R and a model that included clinical status and all identified SVs as covariates [36, 45]. Significant differentially methylated probes between groups were identified at a false-discovery rate (FDR) < 0.05 following multiple test correction by the Benjamini-Hochberg method and were required to show the same direction of change as the NDN cohort’s findings [46]. Further evaluation of potential biological significance was performed using an arbitrary threshold of > 5% mean percent DNA methylation difference between FASD and controls.

### DNA methylation pyrosequencing assay

The bisulfite pyrosequencing assay was designed with PyroMark Assay Design 2.0 (Qiagen; Additional file 1: Table S1). The region of interest was amplified by PCR using the HotstarTaq DNA polymerase kit (Qiagen) as follows: 15 min at 95 °C, 45 cycles of 95 °C for 30s, 58 °C for 30s, and 72 °C for 30s, and a 5-min 72 °C final extension step. For pyrosequencing, single-stranded DNA was prepared from the PCR product with the Pyromark™ Vacuum Prep Workstation (Qiagen) and the sequencing was performed using sequencing primers on a Pyromark™ Q96 MD pyrosequencer (Qiagen). The quantitative levels of methylation for each CpG dinucleotide were calculated with Pyro Q-CpG software (Qiagen).

### The NDN cohort of children with FASD

DNA methylation data from the previous cohort of children with FASD were obtained from GEO (GSE80261) and normalized as described in our original publication [36]. This cohort was collected by NeuroDevNet, a Canadian Network of Centres for Excellence, and is hereby referred to as the NDN cohort [36]. Briefly, we selected the individuals with a confirmed diagnosis of FASD from this dataset, as well as age- and sex-matched typically developing controls, resulting in dataset composed of 83 children with FASD (55 ARND, 18 partial FAS, 10 FAS) and 96 typically developing controls. The mean age (in years) for individuals with FASD was 11.88 and 11.28 for controls, both ranging from 5 to 18 years old. The proportions of males and female differed slightly between groups, with 42 females and 41 males in the FASD cases and 57 females and 39 males in the control group. A skew in self-declared ethnicity was present between the groups, as the majority of controls identified as Caucasian, while the majority of children in the FASD group identified as First Nations. This skew was addressed in the initial epigenome-wide association study through the use of a more ethnically homogeneous subset of the cohort. DNA methylation data were obtained from BEC using the Illumina 450K array and were normalized using the beta-mixture quantile normalization method.

### Cohort of individuals with autism spectrum disorder

Processed DNA methylation data from a publically available dataset of individuals with autism spectrum disorder (ASD) were obtained from GEO (GSE50759). Briefly, this dataset was composed of 48 individuals with ASD and 48 typically developing controls. As per the authors’ description of the GEO data, these were preprocessed using the R packages *minfi* and *sva* to obtain normalized *M* values [47]. The samples consisted of 58 males and 38 females, consistent with the skew towards males in ASD. The mean age (8.84) and range (1–28 years old) differed from the NDN and KBHN studies, and the genetic ancestry of most individuals was Caucasian (European), though a proportion of the cohort was of Nigerian ancestry. DNA methylation data of these samples were obtained from BEC using the Illumina 450K array.

### DNA methylation as a predictor of FASD status

A predictive model of FASD status was created using DNA methylation data and the *caret* package in R [48]. First, a predictive model was created using stochastic gradient boosting on the NDN cohort (83 FASD cases, 96 controls) using the beta-values of the differentially methylated probes identified in the NDN study (648 probes) [36]. The parameters of the modeling were optimized for area under the receiver operating characteristic (ROC) curve by grid tuning for repeated cross-validation (number of trees 50–1500; 1, 5, or 9 interaction depth; 0.1 shrinkage). The optimal model for predicting clinical FASD status using 648 probes was 550 trees, 1 of interaction depth, and 20 minimum observations per node. The KBHN cohort (24 FASD, 24 controls) was then used to verify the predictive sensitivity and specificity of the model. In parallel, 450K data from a cohort of children with ASD were tested to verify the predictive specificity of the model for FASD. The predictor was tested using normalized data that was uncorrected for batch effects to better mimic the potential use of the predictive model by independent groups.

## Results

### The KBHN cohort of children with FASD

As noted, we analyzed genome-wide DNA methylation patterns from 24 children with FASD and 24 typically developing controls, matched for sex and age, ranging from 3.5 to 18 years of age (Table 1). We found that self-declared ethnicity, primary caregiver, and mean age were significantly different between the FASD and control participants (Student’s *t* test; *p* < 0.05). We corrected for the potential effects of age on DNA methylation through the statistical methods outlined below. However, given the confound in self-declared ethnicity and caregiver status, we could not correct for these effects and relied on the previous correction of ethnic bias in the initial NDN study (see below) [36]. Furthermore, we could not account for the different life experiences of individuals with FASD, including potential exposure to adverse early life events at considerably higher levels than those in the general population. It is possible that these distinct experiences in themselves may potentially be associated with DNA methylation patterns.

### Children with FASD and typically developing controls showed differential DNA methylation patterns

Following quality control and normalization, 431,544 sites of the 485,512 sites remained in the final dataset of 48 samples, which were corrected for batch effects using ComBat. While BECs are mostly a homogeneous population of cells, they contain small proportions of CD34- and CD14-positive white blood cells, which can potentially skew DNA methylation analyses. As such, cell type deconvolution was performed to identify any blood contamination in the samples, identifying a trend towards significance in the proportions of different cells types between groups (CD34+, *p* = 0.115; CD14+, *p* = 0.224; BEC, *p* = 0.068). To account for this factor in addition to other potential confounding variables within the dataset, we performed SVA to identify patterns of variation, identifying 6 surrogate variables when protecting the effects of group (FASD vs control). These were correlated with known sources of variation within the data, including cell type proportions and age (Additional file 2: Figure S1).

**Fig. 1:**
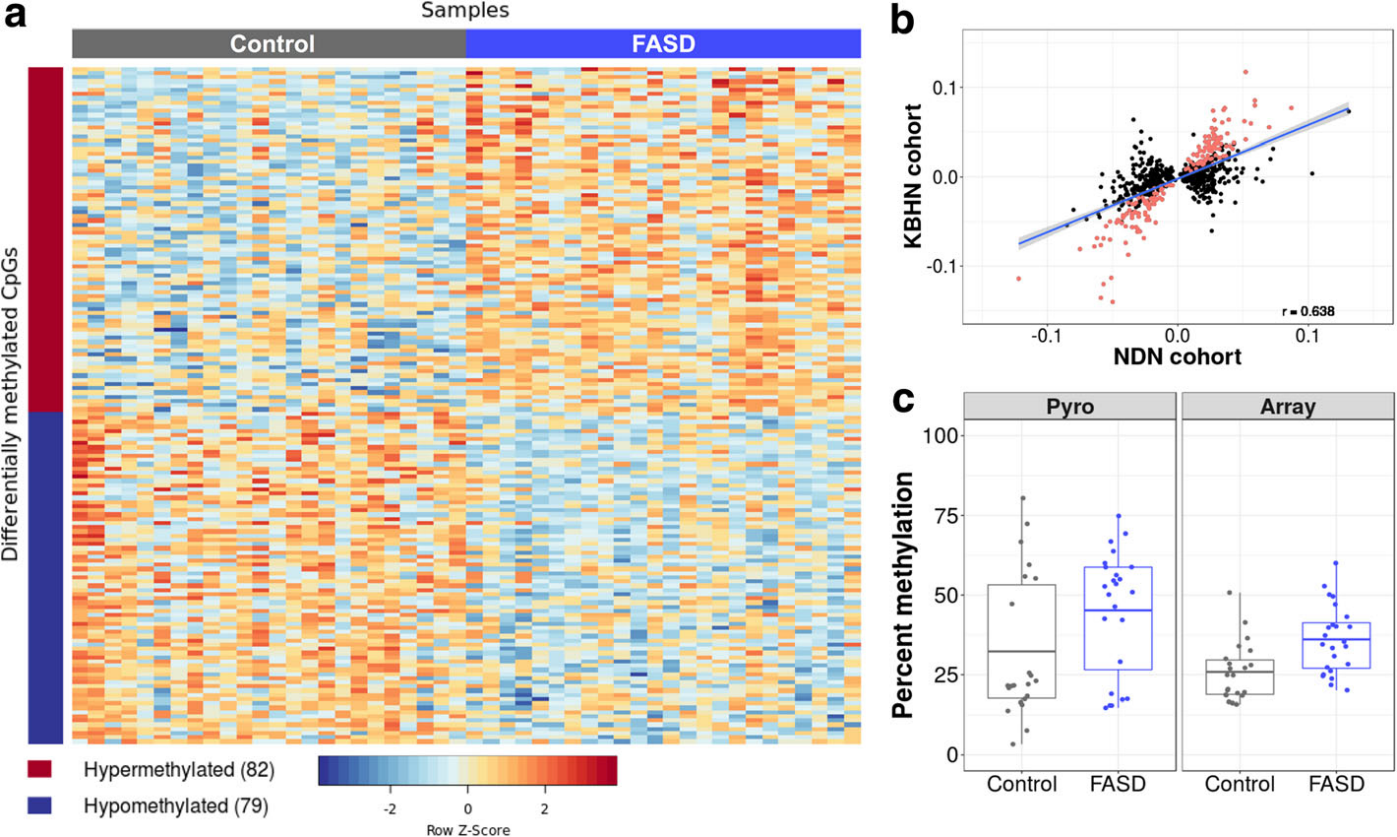
Visualization and verification of the differentially methylated probes. **a** Heatmap of the 161 validated probes validated in the KBHN cohort at an FDR < 0.05 (79 hypermethylated in FASD; 82 hypomethylated in FASD). The percent methylation values (ranging from 0 to 100) were centered, scaled, and trimmed, resulting in a standardized DNA methylation level ranging from − 2 to + 2 (blue-red scale). **b** Scatter plot of the differences in percent methylation between FASD and controls for the 648 differentially probes identified in the NDN cohort. The mean differences between groups were highly correlated between both the NDN and KBHN cohorts (*r* = 0.638, *p* < 2.2e-16). The red points show the probes that were statistically significant (FDR < 0.05) and showed the same direction of change across both studies **c** Verification by bisulfite pyrosequencing in FASD (*blue*) and control (*gray*) samples. The left panel shows the DNA methylation levels from the pyrosequencing assay, while the right panel shows the results from the 450K array. The CpG assayed was located in the CACNA1A gene body (*cg24800175*) and showed statistically significant differences between groups (*p* = 0.04)

**Fig. 2:**
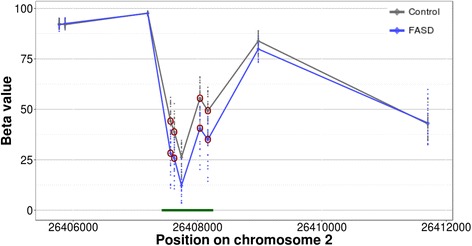
Several differentially methylated CpGs were located in the *Fam59b* gene body. DNA methylation levels for FASD (*blue*) and controls (*gray*) are shown for 10 CpGs within the gene, with the red circles representing the validated hits in KBHN (FDR < 0.05). These were located in a CpG island, illustrated by the green bar at the bottom, which showed an average 13% difference in DNA methylation levels in individuals with FASD versus controls across all five CpGs covered by the 450K array

To identify DNA methylation patterns specific to the FASD group, we coupled differential DNA methylation analysis using a two-group design with the surrogate variables to correct for undesirable variation in the data. Given that we already accounted for ethnicity-related probes as much as possible in the NDN study, it was concluded that the effects of ethnic background would be lessened by using the final 658 differentially methylated CpGs [36]. As such, we performed linear modeling on the probes that were differentially methylated in the first study and remained in the dataset after pre-processing (648 CpGs of 658 from NDN). Of these, 161 CpGs displayed statistically significant differential methylation in the same direction as the initial cohort in the KBHN FASD group compared to the controls at an FDR < 0.05 (Fig. 1a; Additional file 1: Table S2). To assess the probability of validating this many probes, random group subsampling was performed 10,000 times. As none showed more differentially methylated probes than the original replication cohort (maximum = 31 differentially methylated probes), the probability of validating 161/648 probes was < 1e−4 (Additional file 2: Figure S2). Of the 161 validated probes, 82 were up-methylated, while 79 were down-methylated in FASD compared to control samples. Several genes contained multiple differentially methylated CpGs across both cohorts, including *Hla-dpb1* (5), *Fam59b* (4), *Capn10* (3), *Des* (3), *Slc6a3* (3), *Slc38a2* (3), *Fam24a* (2), *H19* (2), and *Tgfb1i1* (2) (Table 2). Moreover, 53 CpGs showed > 5% difference in methylation, an arbitrary cutoff often used to gauge potential biological significance [49]. Three genes contained 2 or more differentially methylated (DM) probes that showed both an FDR < 0.05 and > 5% difference in percent methylation, including *Fam59b* (4 probes), *Hla-dpb1* (2 probes), and *Slc6a3* (2 probes). In particular, the *Fam59b* CpGs were located within a CpG island and showed substantial differences in DNA methylation levels between FASD and control groups, with an average 13% methylation difference across the array probes in the CpG island (Fig. 2). Three additional sites located in intergenic regions showed > 10% percent DNA methylation difference between groups.

**Table 2:** Genes containing multiple differentially methylated CpGs in FASD

Gene	No. of CpGs	Direction of change
*Hla-dpb1*	5	Up
*Fam59b*	4	Down
*Des*	3	Down
*Slc6a3*	3	Up
*Slc38a2*	3	Down
*Capn10*	3	Up
*Fam24a*	2	Up
*H19*	2	Down
*Tgfb1i1*	2	Down

Overall, the percent methylation differences between groups of the 648 analyzed probes were highly correlated between the NDN and KBHN cohorts, as determined by linear modeling (*r* = 0.638, *p* < 2.2e−16; Fig. 1b). We also compared the ranking of probes by *p* value from linear modeling between the NDN and KBHN cohorts; no significant similarities were identified (*p* = 0.91). Of note, 21 of the significant probes with > 5% methylation difference between groups from the NDN study were validated in the present analysis (39 of 41 were present in the filtered KBHN dataset). This proportion (54%) was much higher than all validated probes (25%), suggesting that these potentially represented more robust associations with FASD.

### Bisulfite pyrosequencing verified the differential DNA methylation of CACNA1A

To verify that the differential DNA methylation results did not depend on the method used to measure them, we assessed DNA methylation levels of the cg24800175 probe in CACNA1A. We selected this probe as it was also verified in the initial NDN study, where it similarly showed a > 5% difference in DNA methylation between individuals with FASD and controls. Pyrosequencing results confirmed the DNA methylation levels observed on the 450K array, showing similar DNA methylation levels and differences between groups for CpGs located in *CACNA1A* (Fig. 1c). The Pearson correlation between these two methods was 0.826 and the Bland–Altman plot showed little difference when comparing the 450K array to pyrosequencing, suggesting good concordance between DNA methylation data from the two methods (Additional file 2: Figure S3). Linear regression analysis of pyrosequencing data between FASD cases and controls confirmed differential DNA methylation in this site, even without correcting for covariates (*p* = 0.04).

### DNA methylation patterns classified individuals with FASD versus controls

**Fig. 3:**
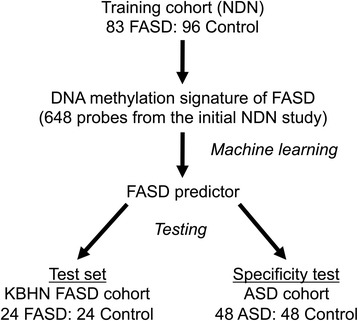
Flowchart of bioinformatic analyses for the DNA methylation predictor of FASD. Briefly, samples from the NDN cohort were used as the training set, and machine learning was performed on the DNA methylation signature of FASD identified in the initial NDN study. The resulting FASD predictor was tested on the KBHN test set, as well as an independent cohort composed of individuals with autism spectrum disorder and typically developing controls to test the specificity of the predictor for FASD

To assess whether DNA methylation data could be used to predict FASD status, we created a predictive algorithm of FASD using machine learning approaches. First, we selected the normalized DNA methylation data (beta-values) of 179 samples from the NDN cohort (83 FASD; 96 control) in the 648 initial probes that were also found in the KBHN data. Our strategy was to build the predictor using an initial training cohort (NDN), followed by subsequent evaluation in the test cohort (KBHN). See Fig. 3 for an overview of steps used to build the FASD predictor.

Using a gradient boosting model in the *caret* package to optimize both sensitivity and specificity (area under the receiver operating characteristic (ROC) curve), we created a predictive model to assess the probability of FASD based on DNA methylation patterns [48]. This method provided weighted values for the different features (CpGs) of the model to determine their importance in classifying the samples. Of the 648 initial features, 183 had non-zero influence on the predictive model and could be used to predict FASD status (Additional file 1: Table S3). As the number of non-zero features was similar to the total number of samples, concerns of model over-fitting were reduced.

**Fig. 4:**
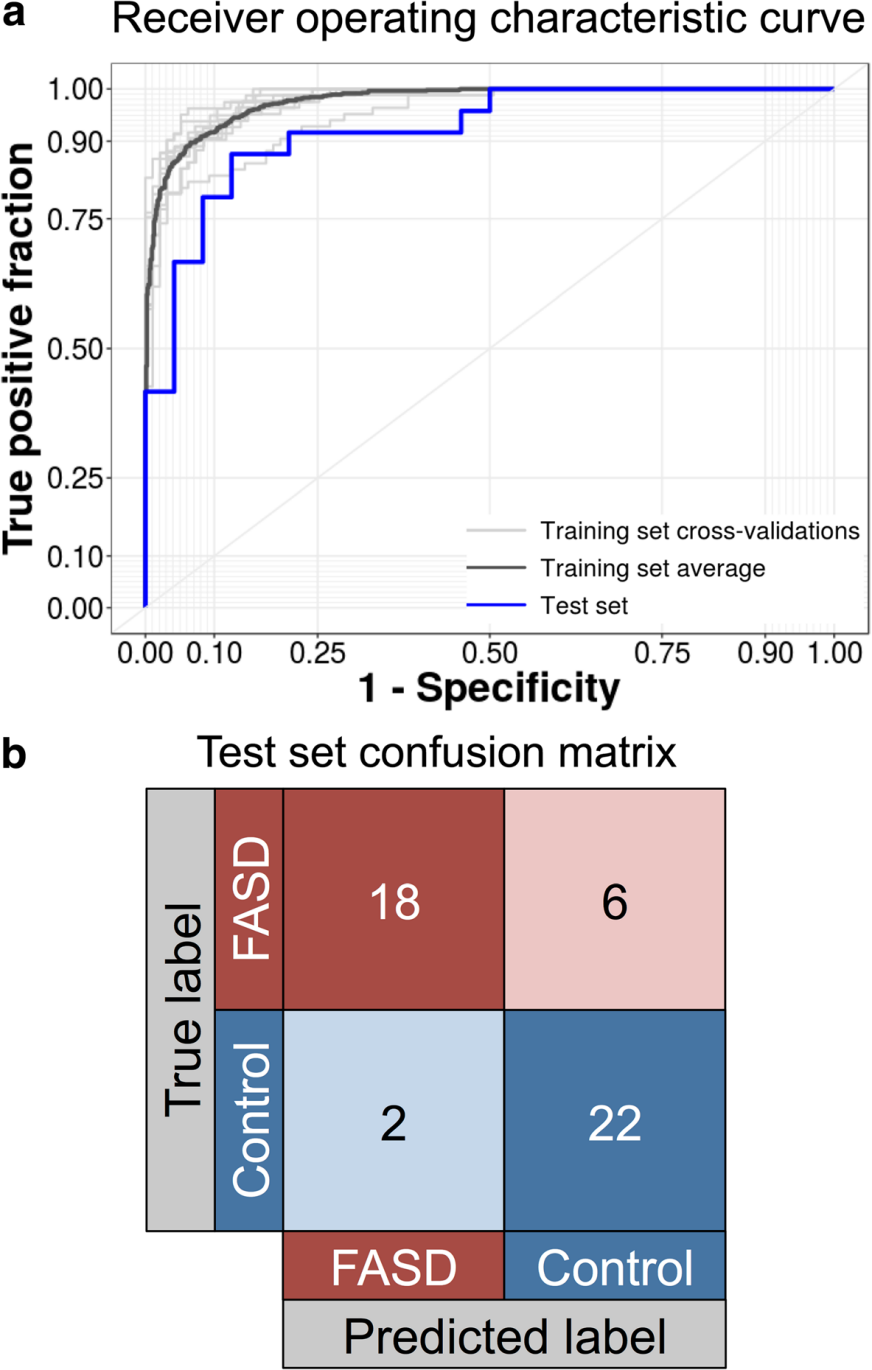
Visualization of the training and test set performance for the DNA methylation predictor of FASD. **a** The DNA methylation predictor created using the 648 probes identified in NDN showed high accuracy in the training cohort (*dark gray*; area under the curve = 0.977) and slightly poorer accuracy in the KBHN test set (*blue*; area under the curve = 0.920). These curves were not significantly different (*p* = 0.192). **b** The confusion matrix displays number of samples classified correctly or incorrectly. Of note, six individuals with FASD in the test set were classified as controls, while only two control samples were misclassified as FASD

Through this approach, the predicted sensitivity and specificity for the training cohort were 0.879 and 0.944, respectively, for an area under the curve of 0.977 (95% confidence intervals, 0.972–0.982; Fig. 4a). The performance of the predictor on the training data indicated that DNA methylation could be used to distinguish FASD cases and controls, although these results will need to be carefully assessed in independent test sets or clinical settings.

To get a better understanding of the utility of this tool, we next assessed the predictive model using the normalized, batch-corrected DNA methylation data of the KBHN cohort as a test set. Of note, these data were not corrected for any covariates or surrogate variables other than batch correction. As expected for analysis of an independent test set, the model performed at a lower level in this cohort, displaying 0.917 sensitivity, 0.75 specificity, and 0.920 area under the ROC curve (Table 3; Fig. 4a). The balanced accuracy of the model in this cohort was 0.833 (95% CI 0.698–0.925), and the ROC curve was not significantly different from the one obtained in the training cohort (*p* = 0.192). Overall, 2 controls were misclassified as FASD and 6 children with FASD were misclassified as controls, giving a negative predictive value (NPV) of 78.6% and a positive predictive value (PPV) of 90%. Given the discrepancies in ethnic backgrounds between FASD and control groups, the misclassified samples were assessed for differences in self-reported ethnicity, caregiver status, age, and buccal cell-type proportions in the classification. We did not identify any skew of these data in the misclassified controls, which were both Caucasian males aged 15 and 16, respectively. Although every misclassified individual with FASD had a previous diagnosis of ARND, a category that was present in high proportion within this cohort, no other patterns emerged between the correctly and incorrectly classified individuals with FASD (3 females/3 males; 3 First Nations/1 Métis/2 Caucasian; aged 6–18). Taken together, these findings suggested that differences in these demographic variables between the groups did not drive their classification.

**Table 3:** Summarized results from the classification algorithm

Training set (NDN)	
AUC	0.977
Accuracy	0.914
Sensitivity	0.879
Specificity	0.944
Test set (KBHN)	
AUC	0.920
Accuracy	0.833
Sensitivity	0.75
Specificity	0.917
False positives	2
False negatives	6
PPV	0.900
NPV	0.786
Negative control (ASD)	
Accuracy	0.990
Sensitivity	NA
Specificity	0.990
False positives	1

### The DNA methylation predictors were not biased by ASD in an independent cohort

BEC samples from an independent published autism spectrum disorder (ASD) cohort were used to assess the specificity of the model in the FASD cohorts. To this end, we used a publically available dataset of 450K array data from the BECs of 48 individuals with ASD and 48 typically developing controls from the gene expression omnibus (GSE50759) [47]. Using processed GEO data from this cohort, the predictor correctly identified the vast majority of individuals in the cohort as non-FASD. The model only misclassified 1 individual as FASD, for a specificity of 0.990 (95% CI 0.943–0.9997), higher than the predicted specificity in the training set. This sample, a 3-year-old female with ASD (51% African ancestry, 41% European ancestry) did not have any particular distinguishing features compared to the correctly classified samples, suggesting that the predictive model was not biased for ASD, sex, age, or African ancestry in this independent cohort.

## Discussion

Epigenetic marks are emerging as potential biomarkers and mediators of environmental exposures, and a growing body of literature suggests that epigenetic factors may be involved in the etiology of FASD. In particular, our recent study using a large cohort of children with FASD to date identified a signature of 658 differentially methylated CpGs in the BEC of individuals with FASD compared to typically developing controls [36]. Here, we present a validation of genome-wide DNA methylation data in a small cohort of individuals with FASD, where we successfully replicated 161 of the 658 differentially methylated CpGs identified in the initial NDN cohort. Furthermore, we demonstrated that DNA methylation data could be utilized to generate a predictive algorithm to classify individuals as FASD or control with high accuracy. These results indicated that DNA methylation in BECs could potentially be used towards developing a screening tool for children at risk for FASD.

Our present findings represent the initial validation of genome-wide DNA methylation differences in individuals with FASD. Of the 161 validated CpGs at an FDR < 0.05, 53 had > 5% difference in DNA methylation levels between groups. This 5% threshold is often used for assessing potential biological relevance in epigenetic studies of psychiatric and neurodevelopmental disorders, and therefore, we confined our interpretation of possible functional implications to CpGs with this effect size [47, 49–51]. Importantly, the biological significance of a 5% difference in DNA methylation is poorly understood, and its functional relevance may be limited in relation to gene expression or cellular physiology. Nevertheless, 21 CpGs showed a > 5% difference between FASD cases and controls at an FDR < 0.05 in both the KBHN and NDN cohorts, suggesting that these may represent the strongest associations with FASD. Although the DNA methylation differences between FASD and controls were highly correlated between the NDN and KBHN cohorts (*p* < 2.2e−16), the majority of CpGs showing the same direction of change did not achieve statistical significance (301/648), potentially due to the replication cohort’s small size or the absence of individuals with only PAE in this cohort. In addition, we verified the results from the 450K array by bisulfite pyrosequencing, confirming the differential DNA methylation results for a CpG located in CACNA1A and supporting that our findings were not an artifact of array technology. As discussed in a recent comprehensive review, we note that the functional relevance of these differences is highly dependent on multiple factors, including subcellular differences, transcription factor binding regulation, density and cooperativity of DNA methylation, or other *cis*-regulatory elements [52].

Several genes previously associated with PAE or FASD contained multiple differentially methylated CpGs with > 5% difference in DNA methylation between groups, including *Fam59b*, *Hla-dpb1*, and *Slc6a3*. The *Hla-dpb1* locus, a member of the major histocompatibility complex proteins, contained several differentially methylated CpGs, which overlapped with a differentially methylated region identified in the NDN study. Given its important function in immune regulation and potential role in rheumatoid arthritis, these differences could potentially reflect some of the immune deficits associated with FASD [53]. Furthermore, the *Fam59b* gene contained several CpGs with substantial (> 10%) differences in DNA methylation levels between individuals with FASD and controls, potentially representing a particularly sensitive locus with regard to FASD. Of note, only one validated CpG was located in one of the protocadherin gene clusters (*Pcdhb18*), which were considerably enriched in previous genome-wide studies of DNA methylation in individuals with FASD [35, 36]. Given that these different studies only showed one overlapping probe, this could indicate higher variability within these gene clusters that may be associated with other variables not present in the current dataset, such as differences in age, body mass index, ethnicity, and socio-economic status.

Of particular interest, we replicated the differential DNA methylation patterns of the two genes involved in dopamine signaling from the NDN cohort, the dopamine transporter *Slc6a3* and the dopamine receptor D4 (*Drd4*). Given the important role of the dopaminergic system in brain development and its interactions with neuroendocrine and immune systems, these differences could potentially reflect broader alterations to signaling pathways in the organism. Of note, the BEC of children exposed to alcohol during prenatal life and breastfeeding also display altered DNA methylation patterns in the promoter region of *Drd4* [34]. Furthermore, several disorders previously associated with allelic variation and DNA methylation in this gene show either overlaps or co-morbidities with FASD, including attention deficit hyperactivity disorder, bipolar disorder, anxiety disorder, schizophrenia, and substance abuse [54–64]. Although it is tempting to interpret these findings in the context of PAE-induced deficits, DNA methylation differences in BEC likely do not fully reflect alterations in the central nervous system. Nevertheless, it has been suggested that BECs may act as a suitable surrogate tissue in human studies of DNA methylation, as they are also derived from the ectoderm [65]. While we did not measure these genes in additional tissues, evidence from animal models suggests that PAE can cause lasting alterations to the epigenome of central nervous system tissues, and as such, these results could potentially represent broader associations with epigenomic patterns in the brain [27].

Although these findings represent the initial validation of genome-wide DNA methylation data in children with FASD, a few particularities of the KBHN cohort limit the interpretability and generalizability of these results. Similar to the original cohort, the KBHN replication cohort was confounded by ethnicity, as the vast majority of FASD cases were from First Nations communities, while controls were mainly Caucasian. Given that genetic background influences DNA methylation patterns, differences between groups may have been, at least partly, due to ethnicity. Unfortunately, the KBHN cohort was too small to separate the groups into more ethnically homogeneous subsets, a method we had previously used to account for ethnicity-related differences in DNA methylation [37]. As such, we performed linear modeling on the sites that had been previously identified in the NDN study, which were partially filtered for ethnicity-related differences during the analysis of the first cohort. However, some of the top differentially methylated genes could potentially be influenced by ethnicity differences between groups in spite of our best efforts. For instance, three known polymorphisms are located within the *Fam59b* locus (dbSNP minor allele frequencies: rs774397935, 1.04%; rs4665833, 5.1%; rs181971256, 21.4%). Although, as of now, none of these are known methylation quantitative trait loci (mQTL), the *Fam59b* gene body contains several mQTLs in the developing human brain, and genetic variation outside the region could potentially influence DNA methylation levels [66]. In addition, nearby genetic variation can also influence DNA methylation patterns in the promoter of *Drd4*, which may be reflected in this cohort through the skew in ethnicity between groups [59]. Although the frequencies of these alleles in First Nation populations have not been assessed, genetic differences between groups could potentially influence DNA methylation levels within this differentially methylated region.

In addition to self-declared ethnicity, significant differences in the primary caregiver were present between groups, as all controls lived with their biological families, while the majority of children with FASD were generally in foster care or adoptive families. While the effects of this disparity on the epigenome are unclear, they could influence DNA methylation patterns through a number of factors, including nutrition, early-life adversity, and socio-economic status [67]. Individuals with FASD also tend to have life experiences different from those of typically developing children, which include early life adversity (e.g., maltreatment or neglect), separation from the biological family/placement in foster care (as occurred in our cohorts), poverty, and familial stress [13, 68]. Importantly, both pre- and postnatal experiences are known to play a role in early programming and thus may also influence DNA methylation patterns. As such, it may be difficult to separate the impacts of PAE and environmental adversity, and studies evaluating FASD may in many instances assess a combination of different factors and exposures, which is often the reality in this population. Nevertheless, our findings demonstrated clear and replicable associations between FASD and DNA methylation patterns across two independent cohorts. We believe that our use of SVA to partially account for unknown factors that could influence DNA methylation reduced some of the potential confounds associated with the cohort design. Future studies with larger groups that are balanced for ethnicity, age, and additional variables, including a focus on environmental stress/adversity, will be necessary to tease out these differences and further validate our findings.

Finally, we show here that DNA methylation patterns can be utilized as predictive variables for FASD in clinical populations. These findings complement and extend previous studies that investigated different molecular and physiological markers to help screen children for potential prenatal alcohol exposure, including alcohol metabolites in mothers and children, circulating miRNA in mothers, and cardiac orienting response in children [69–72]. In particular, eye tracking measures have been used in a small cohort of children to distinguish children with FASD, ADHD, or typically developing controls with relatively good accuracy [73]. In contrast to these studies, we selected only individuals with confirmed FASD from the initial NDN training cohort to create a DNA methylation-based predictor that was specific to individuals with an FASD diagnosis. The classification model was tuned to screen children at a higher risk for FASD with high sensitivity and specificity in an attempt to balance the false-positive and false-negative rates. Importantly, our results suggest that DNA methylation predictors can achieve high accuracy in the classification of individuals with FASD versus controls across multiple cohorts. Moreover, the predictive algorithm appeared to be largely independent of typical confounding factors, such as age, sex, ethnicity, and cell type composition of the samples. The predictor was also unbiased towards individuals with ASD and although there was no report of FASD in this independent cohort, it is possible that our reported false-positive could be due to an undiagnosed FASD case [74]. Collectively, these results support the use of DNA methylation as a potential screening tool for FASD.

## Conclusions

Given the broad spectrum of cognitive, behavioral, and biological deficits associated with PAE, FASD places an important strain on both societal resources and the affected individuals and families. As such, accurate screening methods are necessary to identify children at risk for FASD at an early age, when interventions are most effective. Our findings provide an initial stepping-stone towards epigenetic biomarkers of FASD and could potentially be adapted for the development of related screening tools for neurodevelopmental disorders. Validation of these tools across different cohorts, with increased sample sizes, varying ages, ethnicities, and better documented environmental exposures will be essential to parse out the strongest associations and to develop reliable epigenetic screening tools for FASD.

## Additional files

Additional file 1: Supplementary tables. (XLSX 69 kb)

Additional file 2: Supplementary figures. (DOCX 130 kb)

## Abbreviations

450K array: Illumina HumanMethylation450 BeadChip array
ARND: Alcohol-related neurodevelopmental disorder
ASD: Autism spectrum disorder
BEC: Buccal epithelial cell
CpG: Cytosine-guanine dinucleotide
DNA: Deoxyribonucleic acid
FAS: Fetal alcohol syndrome
FASD: Fetal alcohol spectrum disorder
FDR: False discovery rate
GEO: Gene Expression Omnibus
KBHN: Kids Brain Health Network
NDN: NeuroDevNet
PAE: Prenatal alcohol exposure
PCDH: Protocadherin
RNA: Ribonucleic acid
ROC: Receiver operator characteristic
SV: Surrogate variable
SVA: Surrogate variable analysis
