# Supplementary material for: DNA methylation as a predictor of fetal alcohol spectrum disorder
Source: Clin Epigenetics. 2018 Jan 12;10:5. doi: 10.1186/s13148-018-0439-6 (PMC5767049; doi:10.1186/s13148-018-0439-6)
Supplement: Supplementary file 2 — Supplementary figures. (DOCX 130 kb) [file 13148_2018_439_MOESM2_ESM.docx]

Supplementary figures


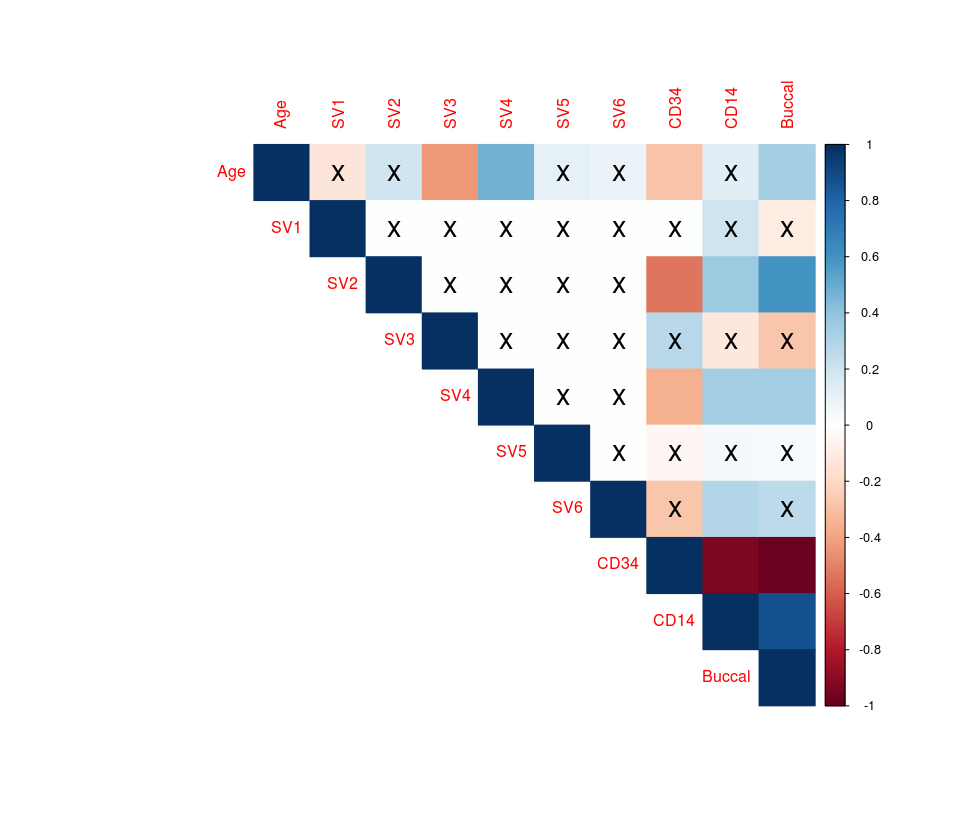


**Figure S5.1. Surrogate variables were correlated with known sources of variation.** Pearson correlations were performed between the 6 surrogate variables identified in the DNA methylation data and known covariates: age and predicted CD34+/CD14+/buccal proportions. SV2 and SV4 were highly correlated with cell type proportions, while SV3 and SV4 were correlated with age, suggesting that these SVs could be used to correct for these known sources of variance in the dataset. Squares with and X were not significant (p>0.05).


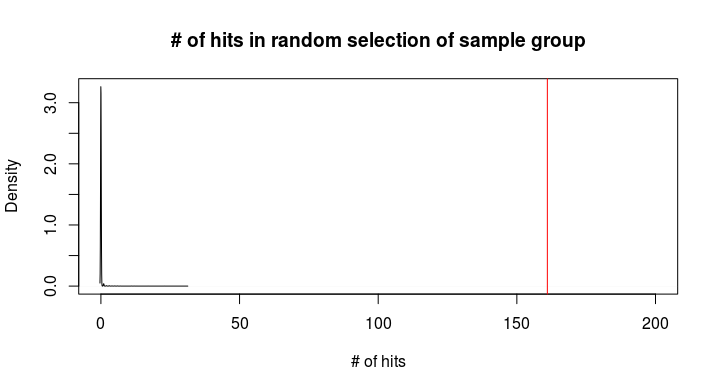


**Figure S5.2. Number of hits in a random selection of sample group.** Random group subsampling was performed 10,000 times to obtain the probability of validating differential methylation at 161/648 probes. As none showed more differentially methylated probes than the original replication cohort (maximum = 31 differentially methylated probes), the probability of validated 161/648 probes was < 1e-4.


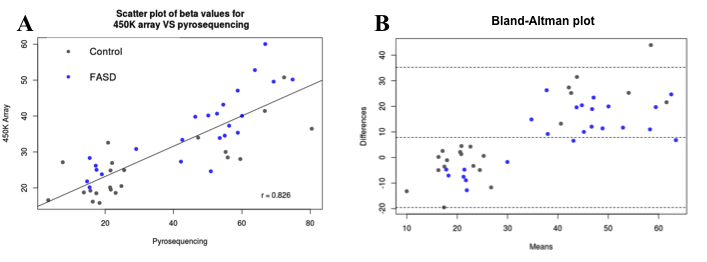


**Figure S5.3. Scatter plot and Bland-Altman plots of bisulfite pyrosequencing and 450K array data.** A) The values for the two methods were highly correlated (p = 0.826) for individuals with FASD (blue) and controls (grey). B) The Bland–Altman plot showed little difference when comparing the 450K array to pyrosequencing, suggesting good concordance between DNA methylation data from the two methods.
